# Cognitive Impairment in Patients with Chronic Neuropathic or Radicular Pain: An Interaction of Pain and Age

**DOI:** 10.3389/fnbeh.2017.00100

**Published:** 2017-06-13

**Authors:** Orla Moriarty, Nancy Ruane, David O'Gorman, Chris H. Maharaj, Caroline Mitchell, Kiran M. Sarma, David P. Finn, Brian E. McGuire

**Affiliations:** ^1^Pharmacology and Therapeutics, School of Medicine, National University of IrelandGalway, Ireland; ^2^Centre for Pain Research, National Centre for Biomedical Engineering Science, National University of IrelandGalway, Ireland; ^3^Division of Pain Medicine, Galway University HospitalGalway, Ireland; ^4^School of Psychology, National University of IrelandGalway, Ireland

**Keywords:** neuropathic pain, radicular pain, age, cognition, attention, memory, executive function

## Abstract

A growing body of empirical research has confirmed an association between chronic pain and cognitive dysfunction. The aim of the present study was to determine whether cognitive function is affected in patients with a diagnosis of chronic neuropathic or radicular pain relative to healthy control participants matched by age, gender, and years of education. We also examined the interaction of pain with age in terms of cognitive performance. Some limitations of previous clinical research investigating the effects of chronic pain on cognitive function include differences in the pain and cognitive scale materials used, and the heterogeneity of patient participants, both in terms of their demographics and pathological conditions. To address these potential confounds, we have used a relatively homogenous patient group and included both experimental and statistical controls. We have also specifically investigated the interaction effect of pain and age on cognitive performance. Patients (*n* = 38) and controls (*n* = 38) were administered a battery of cognitive tests measuring IQ, spatial and verbal memory, attention, and executive function. Educational level, depressive symptoms, and state anxiety were assessed as were medication usage, caffeine, and nicotine consumption to control for possible confounding effects. Both the level of depressive symptoms and the state anxiety score were higher in chronic pain patients than in matched control participants. Chronic pain patients had a lower estimated IQ than controls, and showed impairments on measures of spatial and verbal memory. Attentional responding was altered in the patient group, possibly indicative of impaired inhibitory control. There were significant interactions between chronic pain condition and age on a number of cognitive outcome variables, such that older patients with chronic pain were more impaired than both age-matched controls and younger patients with chronic pain. Chronic pain did not appear to predict performance on the Wisconsin Card Sorting Task, which was used a measure of executive function. This study supports and extends previous research indicating that chronic pain is associated with impaired memory and attention.

**Perspective:** Compared to healthy control participants, patients with chronic neuropathic or radicular pain showed cognitive deficits which were most pronounced in older pain patients.

## Introduction

Chronic pain is a debilitating condition associated with biopsychosocial consequences. Subjective reports by chronic pain patients and objective empirical research have demonstrated that chronic pain is associated with cognitive deficits in various domains of functioning including, attention, working memory, and executive function (Moriarty et al., 2011; Berryman et al., 2013; Moriarty and Finn, 2014). However, the problem of pain-related cognitive impairment remains under-researched due to various methodological barriers (McGuire, 2013).

Research suggests that cognitive deficits occur across a range of pain conditions [e.g., migraine (Meyer et al., 2000; Calandre et al., 2002; Mongini et al., 2005), fibromyalgia (Grace et al., 1999; Park et al., 2001; Luerding et al., 2008), or diabetic neuropathy (Ryan et al., 1992, 1993), but less emphasis has been placed on examining specific pain types (e.g., neuropathic, inflammatory), irrespective of their etiology. One study comparing different types of pain found that attention was impaired to a similar extent in rheumatoid arthritis, musculoskeletal pain, and fibromyalgia patients compared with healthy controls (Dick et al., 2002). Conversely, there is evidence that emotional decision making was impaired in lumbar spinal or radicular pain of the lower back, but not in Complex Regional Pain Syndrome (CRPS; Apkarian et al., 2004a), and general cognitive functioning was worse in neuropathic pain patients than in patients with a diagnosis of mixed neuropathic and nociceptive pain (Povedano et al., 2007). Although previous investigations of cognition in chronic pain have included neuropathic pain patients as part of a wider sample, few studies have examined performance specifically in neuropathic/radicular pain. In one of these studies, Povedano et al. (2007) reported cognitive impairment in a neuropathic pain cohort compared with the normative sample for the Mini Mental State Exam (MMSE). Two limitations of the study were the absence of a matched comparison group, and the reliance on the MMSE, which may not detect subtle deficits in particular cognitive domains.

Increasing age is consistently associated with cognitive decline (Salthouse, 1996; Salthouse et al., 1998), and there is evidence to suggest that age may moderate the impact of pain on cognitive performance in human and animal models (Leite-Almeida et al., 2009; Oosterman et al., 2013). Based on the hypothesis that pain competes for available attentional resources (Eccleston, 1994; Eccleston and Crombez, 1999), it could be predicted that the negative effect of pain on cognitive function would exacerbated as cognitive function declines with age. However, a positive relationship between reported pain ratings and executive function has also been demonstrated in elderly populations with Alzheimer's disease or arthritis/arthrosis (Scherder et al., 2008; Oosterman et al., 2009). This may simply suggest that pain report is less reliable in the case of more severe cognitive decline, but demonstrates that age is an important determinant of the relationship between pain and cognitive function, and thus requires further investigation.

The aim of the present study was to investigate the effects of pain on cognitive functioning, which would address, where possible, the limitations associated with previous research. Therefore, the study was designed to investigate cognitive functioning specifically in patients with chronic neuropathic or radicular pain, to probe performance in a variety of cognitive domains by exposing participants to a comprehensive battery of well-validated cognitive tests, and to minimize potential confounds. These include differences in the pain and cognitive scale materials used, and the heterogeneity of patient participants both in terms of their demographics and pathological conditions.

Our specific hypotheses were that: (1) chronic neuropathic/radicular pain patients would demonstrate impairments in cognitive performance compared with healthy participants even after controlling for potentially confounding factors, for example, levels of education, affective state and nicotine, alcohol and caffeine consumption. (2) We expected that in models predicting the effect of pain on cognitive performance, a pain × age interaction would emerge whereby older individuals with chronic pain would display the greatest levels of impairment; and (3) that in pain patients, cognitive performance would be predicted by the severity of their pain.

## Materials and methods

### Participants

A total of 38 chronic pain patients and 38 control participants took part in the study. Patients with chronic neuropathic pain or radiculopathy (minimum of 3 months, diagnosed by a specialist pain physician) were identified from the database of patients attending a tertiary pain management clinic at Galway University Hospital, Galway, Ireland. Recruitment of control participants was achieved through placement of advertisements in public places and in the local and national print media. Exclusion criteria were: age < 18 years; self-reported diagnosis of pre-existing cognitive impairment or major psychiatric illness (including major depressive disorder or generalized anxiety disorder); self-reported history of substance abuse, diabetes, epilepsy, seizures, or traumatic brain injury; and in the case of control participants, self-reported history of chronic pain. Patient and control groups were matched by gender, age, and education (Table 1). All participants gave informed written consent, in person, prior to the test session, and all testing procedures were carried out at University Hospital Galway or National University of Ireland, Galway. The study received full institutional approval from the Research Ethics Committee of the National University of Ireland, Galway and from the Galway University Hospitals Research Ethics Committee.

**Table 1:** Demographic information.

	**Chronic pain**	**Control**
	**Total (Male, Female)**	**Total (Male, Female)**
Total number of participants	38 (16, 22)	38 (16, 22)
	**Mean (standard deviation)**	**Mean (standard deviation)**
Age (years)	45.6 (9.9)	44.2 (10.4)
Duration since last consumption of:		
Nicotine (h)	1.6 (3.5)	3.1 (6.6)
Caffeine (h)	5.3 (10.7)	5.8 (8.3)
Years of Education	13.8 (3.8)	15.2 (3.0)
	**Total aged** ≥ **45 years/**<**45 years**	**Total aged** ≥ **45 years/**<**45 years**
Number of participants in each age classification	22/16	17/21
	**Percentage of participants in group**	**Percentage of participants in group**
% of participants who smoke	50.0	18.4^**^

** *p < 0.01*.

### Procedure

Patient participants were sent (by post) an information sheet and an invitation to participate in the research study, at least 1 week in advance of the assessment. To reduce patient burden, chronic pain patients were invited to complete the assessment on the day of a routinely scheduled appointment at the pain clinic. The testing was completed in advance of the clinic appointment to avoid the potentially confounding effects of interventional analgesic treatments. Where patients could not attend on the day of their appointment, but consented to participate, the assessment was scheduled for an alternative date. Medication status at the time of the assessment was not evaluated. For control participants, an outline of the study was provided in the public advertisements, and each participant was provided with a more detailed information sheet by the examiner prior to consenting to take part in the study.

### Demographics

Demographic information, including age, gender, and number of years of education, was collected for all participants using a standard form. In addition, participants were asked to estimate, if applicable, the length of time since they had last consumed nicotine and/or caffeine.

### Pain assessment

Participants in the patient group were asked to complete the Chronic Pain Grade (CPG) Questionnaire (Von Korff et al., 1992). This 7-item scale provides measures of current pain and pain over the previous 3 months, as well as a measure of pain-related disability and an overall chronic pain grade classification, with responses recorded on a 10-point Likert scale. The CPG has been validated as a self-completion measure and is widely used in chronic pain research (Elliott et al., 1999; Dunn et al., 2008; Raftery et al., 2011). In addition, pain patient participants were asked to indicate painful areas on a manikin diagram and to estimate the number of months since the diagnosis of their pain. The total number and drug classification of patients' analgesic medications were also recorded.

### Perceived impact of pain

Patients' subjective ratings of the perceived impact of pain on cognitive function (concentration, memory, problem solving, and decision making) were recorded on a 10-point Likert scale where 0 was “no interference” and 10 was “extreme interference.” This single-item scale was developed by the research team and was phrased in a manner similar to other CPG “interference” items (“In the past 3 months, how much has pain interfered with your concentration, memory, problem solving or decision making?”).

### Depressive symptoms and state anxiety

Depressive symptoms were assessed using the 9-item Patient Health Questionnaire (PHQ-9) depression scale (Kroenke et al., 2001). This questionnaire is based on the Diagnostic and Statistical Manual of Mental Disorders-IV criteria, and has been widely used in research (Kroenke et al., 2001; Kroenke and Spitzer, 2002; Lowe et al., 2004). Scores were computed to give a tentative diagnosis of depression and a measure of symptom severity. The questionnaire responses also gave an indication of symptom-related functional impairment (ability to “work, take care of things at home or get along with other people”). The level of anxiety at the time of cognitive testing was measured using the “state” portion of the State-Trait Anxiety Inventory (STAI), Form Y (Spielberger et al., 1983). The STAI-S (20 items) provides a measure of “state” or current anxiety at the time of completing the questionnaire.

### Neuropsychological tests

#### General intellect

An estimate of participants' intelligence quotient (IQ) was obtained using a two-test short-form of the Wechsler Adult Intelligence Scale-III (WAIS-III, Wechsler, 1997a), the Digit-Symbol Coding and Information subtests. Estimated full-scale IQ obtained using this dyadic short form of the WAIS-III has been shown previously to correlate (*r*^2^ = 0.82) with IQ-values obtained using the full 12-subtest scale (Sattler and Ryan, 2001), and this combination of subtests was chosen based on its relatively short administration time. In the Digit-Symbol Coding subtest, participants were presented with nine digit-symbol pairs followed by a list of digits only. Participants were required to fill in the symbol corresponding to each digit as quickly as possible on a standard form. For the Information subtest, the participant was required to answer a series of factual general knowledge questions about common events, objects, places, and people. Raw scores for both subtests were converted to age-adjusted scaled score equivalents using the WAIS-III Administration and Scoring Manual (Wechsler, 1997a). Full-scale IQ was then estimated from the sum of the scaled scores using the extrapolation tables taken from Sattler and Ryan (Sattler and Ryan, 2001).

#### Verbal memory

The Logical Memory subtests I and II of the Wechsler Memory Scale-III (WMS-III, Wechsler, 1997b) were used to assess short- and long-term verbal memory and recognition memory. Two different stories (A and B) were read to the subject, and immediately afterwards the subject was asked to retell the story from memory.

After an interval of ~25 min, the participant was again asked to recall as many details as possible from both stories A and B. For recognition memory, the participant was required to give “Yes” or “No” answers to a set of 30 questions relating to stories A and B, which includes a mixture of correct and incorrect statements about the story content. Participants were scored on the accuracy of the story recall (“story” and “theme” units) and number of correct responses to recognition questions. Raw scores were converted to age-adjusted scaled score equivalents using the WMS-III Administration and Scoring Manual (Wechsler, 1997b).

#### Spatial memory

The spatial span subtest of the WMS-III was used to measure short-term spatial memory capacity. The test was administered using the spatial span board, which consists of 10 cubes with the numbers 1–10 printed on the sides of the cubes facing the examiner. For spatial span forward, the examiner tapped the cubes in a specified sequence pattern and then asked the participant to tap the same sequence. For spatial span backward (reverse), the participant was asked to tap the sequence in reverse order. The sequence length increased until the participant could no longer replicate the sequence correctly. Raw scores were converted to age-adjusted scaled score equivalents using the WMS-III Administration and Scoring Manual (Wechsler, 1997b).

#### Attention/vigilance and psychomotor speed

The Continuous Performance Test—Identical Pairs (CPT-IP), adapted from the MATRICS (Measurement and Treatment Research to Improve Cognition in Schizophrenia) Consensus Cognitive Battery (Nuechterlein and Green, 2004), was used to measure attention and vigilance. This computerized test required the participant to monitor a series of 4-digit numbers as they appeared briefly on the computer screen. The participant responded to the sequential appearance of identical pairs of numbers by clicking a mouse as quickly as possible. The number of correct responses, the number of incorrect responses and the reaction time were recorded automatically. Incorrect responses were categorized as “false alarms” (i.e., responses to similar, but not identical, numbers presented in sequence) or random responses. The Dprime (range 0–4.2), an indication of the rate of hits to false alarms, was also calculated as an index of attention. The average reaction times to both hits and false alarms during the CPT-IP trial were used as tentative measures of psychomotor speed.

#### Executive function

Executive function was assessed using the Wisconsin Card Sorting Test—Computerized Version 4 (WCST-CV4). This test measured the respondent's ability to adapt to changing schedules of reinforcement (i.e., the “rules” about the task) and thus assesses cognitive flexibility, a key component of executive functioning (Berg, 1948). The computerized version used in this study presented the participant with four key cards and a stimulus card. The cards were matched according to one of three categories: color, shape or quantity of items on the card. Matching is achieved by placing the cursor on the key card selected and left clicking the mouse. The participants were not told how to match the cards, but they were given verbal and visual feedback on whether each match was “right” or “wrong.” The category by which the cards were to be matched changed without warning after presentation of every ten stimulus cards; the subject was required to recognize the changed “rules” and identify the new presentation pattern. Raw scores and demographically (age and education) corrected standard scores were computed for five outcome measures: percentage errors, percentage perseverative (repetitive) responses, percentage perseverative errors, percentage non-perseverative errors and, percentage conceptual level responses. Raw scores were also computed for the number of categories completed, the number of trials to complete the first category, number of failures to maintain set and a learning-to-learn score (indicative of conceptual efficiency across consecutive categories).

#### Statistical design and analysis

Data were analyzed using the Statistical Package for the Social Sciences (SPSS, version 18, IBM Corp., USA) for Windows. The statistical analyses were performed in two distinct phases; the first included both patient and control participant data, while the second included patient data only.

#### Analysis by pain condition (phase I)

In this phase of analysis, hypotheses (1) and (2), as stated in the Introduction, were tested. The first level of analysis was the computation of descriptive and inferential statistics. This was followed by correlation analyses of cognitive outcome measures and participant characteristics aimed at identifying potential covariates. The variables included in the correlation analyses were: age, gender, number of years of education, smoking status and time since last nicotine consumption, time since last caffeine consumption, and depression and anxiety scores. These variables can predict cognitive performance (see for example, review by Moore et al., 2009), effect of alcohol (Rohrbaugh et al., 1988), nicotine (Foulds et al., 1996; Mancuso et al., 1999), and caffeine (Brice and Smith, 2001; Kelemen and Creeley, 2001). We intended controlling for correlates of the outcomes when testing our hypotheses. This would allow us to examine the extent to which the hypothesized effects emerged, having held constant important correlates of the outcome being investigated.

Hierarchical multiple regression analyses were used to test the hypotheses; groups were coded as −1 (patient group) and +1 (control group), age was mean-centered, and an interaction term [group (−1/+1) × age (mean-centered)] was calculated. The purpose of centring was to minimize multicollinearity (arising, in this case, from entering both the variables and their interaction into the regression equation). Participant-characteristic variables that were correlated with the cognitive outcomes were entered into the first block of the regression model. Values for significance levels and β coefficients quoted in the text are for the overall model. Interaction plots for estimated marginal means (2 × 2 ANCOVA with patient vs. control and age median-split at 45 years) were used for visualization of the directionality of effects and for determining the nature of the interaction between variables (data not shown).

#### Analysis by pain variable (phase II)

The second phase of the study tested hypothesis (3), as stated in the Introduction. As in Phase I, an interaction effect between pain variables and age was also hypothesized, and this was tested in the analyses. The pain variables investigated were present pain intensity, pain-related disability score, number of painful areas, and pain chronicity (number of months since diagnosis of pain). Additional patient characteristics investigated as potential covariates were the presence or absence of medication (and different medication sub-groups), total number of medications, percentage pain relief from medications, and self-assessed impairment of cognitive function. Descriptive and inferential statistics relating to pain information (for patient participants only, *n* = 38) were calculated. Correlation matrices, using the patient group data only, were constructed to identify potential covariates. Each of the pain variables was centered, and interaction terms with mean-centered age were calculated. Hierarchical regression analyses were again used to test the hypothesis.

## Results

### Participant characteristics

Demographic and psychological descriptors of pain patient and control groups are presented in Tables 1, 2. Assumptions of the relevant tests were checked using a range of graphical (e.g., box plots, normality plots) and inferential tests of normality. Basic between-group comparisons were used to test the extent to which the matching procedure was successful. There were no significant differences between patient and control groups in age, gender, years of education, or duration since last consumption of caffeine or nicotine. There were, however, significantly more smokers in the patient group than in the control group, and patients exhibited higher depressive-symptom scores, increased depression-related functional impairment and greater levels of state anxiety than controls (see Tables 1, 2).

**Table 2:** Psychological variables.

	**Chronic pain**	**Control**
	**Mean (Standard deviation)**	**Mean (Standard deviation)**
**STATE ANXIETY**		
SAI total score	42.84 (12.48)	28.58 (7.40)^***^
**DEPRESSION**		
Depression severity score	13.30 (5.13)	1.79 (2.20)^***^
	**Percentage of participants in group**	**Percentage of participants in group**
% of participants with clinically relevant depression scores^†^	50.0	0^***^
% of participants with a “major” depressive disorder classification^††^	36.8	0^***^
% of participants with depression-related functional impairment^†††^	86.8	18.9^^***^^

† *Clinically relevant depressive symptoms were considered if the participant's answers fell within the highlighted section of the 9-item Patient Health Questionnaire (PHQ-9) on four or more items, one of which corresponded to items 1 or 2*.

†† *The putative type of depressive disorder was classified as “major depressive disorder” depending on the number of items answered in the highlighted section of the PHQ-9*.

††† *Depression-related functional impairment was assessed by item 10 of the PHQ-9, and deemed present if the item was endorsed as “somewhat difficult” or greater*.

*** *p < 0.001*.

### Cognitive variables

#### Analysis by pain condition (phase I)

Prior to testing the hypothesized effects, group differences (patient vs. control) were explored. Results indicated consistently lower outcome scores in the patient group, or higher scores on reverse scales (number of false alarms and number of random responses in the CPT-IP, and raw scores for % errors, % perseverative responses, % perseverative errors, % non-perseverative errors, number of trials to complete the first category and failure to maintain set in the WCST, see Table 3). Significant differences between groups, where observed, are indicated in Table 3.

**Table 3:** Group comparisons of cognitive outcomes.

	**Chronic pain**	**Control**
	**Mean (Standard deviation)**	**Mean (Standard deviation)**
Estimated full scale IQ	87.61 (14.83)	103.16 (13.10)^***^
**VERBAL MEMORY**		
**Immediate**		
Unit Recall	8.61 (3.10)	11.58 (2.77)^***^
Theme Recall	8.45 (3.11)	10.76 (2.42)^**^
Learning Slope	3.97 (2.73)	4.89 (2.70)
**Delayed**		
Unit Recall	9.24 (2.60)	11.24 (2.66)^**^
Theme Recall	9.24 (2.74)	10.58 (2.89)
Recognition	25.11 (3.14)	26.37 (2.30)
% Retention	77.78 (13.31)	77.34 (15.10)
**SPATIAL MEMORY**		
Forward	8.42 (2.94)	9.87 (3.35)^*^
Reverse	9.42 (3.59)	11.03 (2.81)^*^
Total	8.66 (3.40)	10.45 (3.06)^*^
**ATTENTION**		
Hits	22.16 (3.99)	23.50 (3.90)
False Alarms	8.42 (5.20)	6.47(3.70)
Randoms	3.34 (4.10)	2.11 (2.893)
CPT D Prime	1.11 (0.38)	1.29 (0.30)^*^
**PSYCHOMOTOR SPEED**		
Hits reaction time (s)	544.38 (75.34)	559.59 (665.40)
False alarm reaction time (s)	562.72 (112.69)	566.51 (126.00)
**EXECUTIVE FUNCTION (RAW SCORES)**		
% Errors	35.42 (19.01)	28.95 (18.42)
% Perseverative Responses	21.50 (18.25)	16.03 (14.31)
% Perseverative Errors	18.87 (13.74)	14.11 (11.07)
% Non-perseverative Errors	16.61 (10.50)	14.82 (10.81)
% Conceptual Level Responses	52.76 (27.69)	63.16 (25.36)
Categories Completed	3.63 (2.59)	4.63 (2.12)
Trials to First Category	38.37 (44.64)	31.03 (37.34)
Failure to Maintain Set	0.95 (1.16)	0.63 (1.15)
Learning to Learn	0.15 (6.18)	0.86 (4.10)
**EXECUTIVE FUNCTION (STANDARD SCORES)**		
% Errors	86.05 (15.86)	91.00 (17.81)
% Perseverative Responses	89.92 (20.34)	95.03 (19.50)
% Perseverative Errors	88.74 (19.68)	94.79 (19.22)
% Non-perseverative Errors	88.18 (16.58)	90.34 (15.76)
% Conceptual Level Responses	84.84 (16.71)	90.84 (18.14)

* *p < 0.05*,

** *p < 0.01*,

*** *p < 0.001*.

A correlation matrix of participant characteristic and cognitive outcome variables was used to identify potential covariates (see Table S1). Gender and smoker/non-smoker classifications were correlated with a number of cognitive measures (gender correlated with immediate verbal memory and reaction time; cigarette smoking correlated with immediate verbal memory), while, as expected, a large number of the outcomes were positively correlated with years of education and negatively correlated with measures of depression and state anxiety. Interestingly, the duration since last consumption of caffeine was positively correlated with scores measuring attentional performance. Thus, an increase in the time since participants had last consumed caffeine was associated with higher scores, an effect inconsistent with the recognized acute effect of caffeine as a CNS stimulant (Brunye et al., 2010).

The hypotheses predicted an effect of participant group (patient or control) and an interaction effect of group and age, and were tested through a series of individual hierarchical regressions for each of the cognitive outcomes. Significant covariates of the dependent variable (identified by correlation analysis) were entered in the first block, group (patient vs. control, coded −1/+1) and age (mean-centered) were entered in block 2, and the interaction term (pain × age) was entered in block 3. Statistics for cognitive variables predicted by group or by the interaction term (at levels close to or below the *p* = 0.05 level of statistical significance) are presented in Table 4.

**Table 4:** The contribution of pain, pain-age interaction and other factors to cognitive outcome measures.

	**Estimated FSIQ**	**Verbal learning slope**	**Verbal recognition memory**	**Spatial span reversal**	**CPT hit responses**	**CPT false alarm responses**	**CPT random responses**	**CPT hit reaction time**
**BLOCK 1**								
Years of education (β)	0.37^***^	0.07	–	0.23	0.07	−0.22^*^	−0.17	–
Last caffeine (β)	–	–	–	–	–	−0.11	–	–
Gender (β)	–	–	–	–	–	–	–	−0.21
PHQ severity score (β)	−0.003	–	–	–	−0.59^**^	–	–	–
SAI total score (β)	−0.124	−0.27	−0.23	–	–	–	–	–
**BLOCK 2**								
Pain condition (β)	0.33^*^	0.021	0.08	0.21^#^	−0.36^†^	−0.14	−0.12	0.12
Age (β)	−0.16	−0.29^*^	−0.27^**^	0.14	−0.36^**^	0.28^*^	0.36^***^	0.15
**BLOCK 3**								
Pain condition × age (β)	0.03	0.20^+^	0.30^**^	0.01	−0.04	−0.25^*^	−0.20^†^	0.24^*^
Total *R*^2^	0.45	0.19	0.21	0.11	0.26	0.29	0.25	0.16

*FSIQ, Full Scale Intelligence Quotient; CPT, Continuous Performance Task; PHQ, Patient Health Questionnaire; SAI, State Anxiety Inventory*.

*** p ≤ 0.001;

** p ≤ 0.01;

* p ≤ 0.05;

† p = 0.06;

# p = 0.07;

+ *p = 0.08*.

In the regression model predicting estimated full scale IQ (FSIQ), years of education, PHQ-9 severity score and STAI-S total score were entered as controls in block 1. PHQ severity score and STAI-S total score were found to be correlated; however, the observed variance inflation factors (VIFs) were <10, suggesting that the assumption of minimal multicollinearity was not violated and that scores can be assumed to reflect independent constructs. Years of education significantly contributed to the FSIQ model (β = 0.37, *p* < 0.001), as did the presence of pain (β = 0.33, *p* = 0.04). Interaction plots showed that FSIQ scores were decreased in the presence of pain. Neither age nor the interaction term made any significant contribution to the model (see Table 4).

Immediate verbal memory was measured as story-unit and theme-unit recall scores and a learning-slope score. Presence of a chronic pain diagnosis, age, and their interaction term made no contribution to the story-unit or theme-unit regression models. There were no significant predictor variables of the theme-unit model, and only gender significantly contributed to the story recall unit model (β = 0.24, *p* = 0.04). In the case of learning slope, the contribution of STAI-S total score (entered in the first block) approached significance (β = −0.27, *p* = 0.06). There was a significant contribution of age (β = −0.29, *p* = 0.02); the interaction term of pain and age was also associated with an effect close to statistical significance (β = 0.20, *p* = 0.08, Table 4), with interaction plots suggesting that older pain patients performed worse than controls of a similar age and worse than younger pain patients.

Delayed verbal memory was also measured with story-unit and theme-unit recall scores, as well as with a recognition-memory index and with percentage retention. In a manner similar to the case of immediate verbal memory, neither pain condition, age, nor their interaction significantly contributed to either story-unit or theme-unit models, and none of the additional predictor variables significantly affected these models. Furthermore, no significant predictors of percentage retention were identified (though the effect of age was close to significance: β = −0.21, *p* = 0.08). Recognition memory, however, was significantly predicted by both age (β = −0.27, *p* = 0.01) and the pain-age interaction term (β = 0.30, *p* = 0.006, Table 4), with poor performance particularly evident in older pain patients.

Spatial memory was assessed in forward and reverse trials of the spatial span task. Total spatial span and forward trial scores were not predicted by any of the three main independent variables of interest (pain, age, pain × age interaction), or by any of the control measures entered into the model. Effects close to the level of statistical significance were associated with the presence of pain (β = 0.21, *p* = 0.07) and years of education (β = 0.23, *p* = 0.06) in the reverse scores model (Table 4).

Attention was measured using the CPT, the outcome variables of which were the numbers of hits, false alarms and random responses. The number of hits in the CPT was significantly predicted by age (β = −0.36, *p* = 0.002) and there was also a trend for a contribution of chronic pain (β = −0.36, *p* = 0.06, Table 4). The control variable of depressive-symptom score (PHQ severity) was also a significant contributor to the model (β = −0.59, *p* = 0.002, Table 4). The slopes of the interaction plot indicated a reduced number of hits in the control group compared with the patient group; however, the group means in fact indicate an increased number of hits in the control participants compared with the patient group. Further analysis revealed a strong negative association between PHQ severity score and pain condition (*r*^2^ = −0.83, *p* < 0.001). Therefore, despite tolerance and VIF-values being within accepted limits, the relationship between PHQ severity score and pain condition may have affected the overall predictive capability of the model. Age (β = 0.28, *p* = 0.02) and the age × pain interaction term (β = −0.25, *p* = 0.02) made significant contributions to the regression model for number of false alarms (Table 4). The number of random responses was influenced by age (β = 0.36, *p* = 0.001) and there was a near-significant effect for the age × pain interaction term (β = 0.20, *p* = 0.06, Table 4). In the case of the model fitted for D-prime scores, age (β = −0.34, *p* = 0.003) and PHQ severity scores (β = −0.33, *p* = 0.07) emerged as predictors. There were no significant effects of pain or pain-age interaction.

The reaction times to both hits and false alarms in the CPT task were considered measures of psychomotor speed. There were no main effects of age or pain. Though the individual contributions of age and presence of pain were non-significant, the pain-age interaction term significantly contributed to the model for “hit” reaction time (β = 0.24, *p* = 0.03, Table 4). This interaction effect was approaching significance in the case of “false alarm” reaction time (β = 0.21, *p* = 0.08), though the overall model was not significant. The interaction plots suggest that reaction time increased with the presence of pain in the younger participants but was reduced in the presence of pain in the older group.

The independent variables of pain, age, and age × pain interaction did not predict any of the WCST standard scores or the secondary outcomes (number of categories completed, number of trials to first category, failure to maintain set and learning-to-learn score). The only significant predictor was age (number of categories completed: β = − 0.28, *p* = 0.02), whereby older people achieved lower scores than younger participants.

#### Analysis by pain variable (phase II)

Table 5 shows descriptive statistics for variables unique to the pain patient group and Table 6 provides an overview of patients' medication status. Seventy-nine percent of patients were receiving long-term medication for the treatment of pain. Opioids were the most commonly prescribed class of medication, followed by anticonvulsants. The “other” category included classes of analgesics taken by a small number of participants within the sample. These treatments included: antispasmodics, benzodiazepines and other sedative hypnotics, local anesthetics, and transient receptor potential vanilloid 1 (TRPV1) ligands (capsaicin), as well as adjunctive therapies such as acupuncture and spinal-cord stimulation. In cases where a drug had more than one indication (for example, benzodiazepines used as a muscle relaxant, as an anxiolytic, or as treatment for insomnia), the precise reason for which it was prescribed was not queried. More than half (55%) of the patient participants were taking three or more different drugs to manage their pain. The average subjectively-reported relief provided by pharmacological treatments was 37%.

**Table 5:** Pain variables descriptives.

**Pain variable**	**Mean (Standard deviation)**
Present pain intensity	5.63 (3.63)
Average pain intensity	73.16 (13.67)
Pain-related disability score	79.39 (15.12)
Chronic pain grade	3.53 (0.65)
Number of painful areas	5.87 (3.63)
Pain chronicity (months)	101.45 (86.43)
Self-assessed effect of pain on cognition	6.45 (2.74)

**Table 6:** Breakdown of patient medications.

**Medication variable**	**Percentage**
% receiving medication	78.9
% receiving opioids	63.2
% receiving anticonvulsants	42.1
% receiving antidepressants	31.6
% receiving NSAIDs	39.5
% receiving other medication	39.5
	**Mean (Standard deviation)**
% reported pain relief from medications	36.59 (27.04)
Total number of medications	2.7895 (2.04223)

A correlation matrix of cognitive outcome variables and general participant characteristics was again constructed to identify significant correlations to be entered as control variables in the regression models (patient data only). Similarly to Phase I, years of education correlated with a large proportion of the outcome measures (see Table S2). The only other significant correlations were between: duration since last consumption of nicotine and the spatial span forward trial; depression score and number of hits, false-alarm reaction time and WCST failures to maintain set; and STAI-S total score and false alarm reaction time. Correlations between cognitive outcomes and patient-specific variables, including medications and patients' subjective assessment of the effect of their pain on cognitive function were also investigated (see Table S3). Anticonvulsant treatment was found to correlate significantly with learning-slope performance (*r*_pb_ = 0.37, *p* < 0.05) suggesting lower scores in those receiving anticonvulsant medications. On the other hand, antidepressant treatment was associated with higher scores on the attention task (*r*_pb_ = −0.38, *p* < 0.05 D-prime) and improved WCST performance as indicated by a decrease in the number of trials required to complete the first category (*r*_pb_ = 0.34, *p* < 0.05). Self-assessment of the effect of pain on cognitive function was positively correlated with the failure to maintain set in the WCST (*r*_pb_ = 0.45, *p* < 0.01). All significant correlations were entered as control variables in the hierarchical regression analyses. The hypothesis stated that pain variables, and the interaction of these variables with age, would predict differences in cognitive outcomes, and this was again tested through a series of hierarchical regressions. Significant covariates of the dependent variable were entered in the first block. Each pain variable (present pain intensity, average pain intensity, pain-related disability score, pain chronicity and the number of painful areas) was tested individually. Both the pain variable and age were centered and entered in block 2, and the interaction term was entered in block 3.

Pain chronicity was found to contribute significantly to the models of FSIQ (β = 0.31, *p* = 0.05), immediate verbal memory (β = 0.47, *p* = 0.003) and delayed verbal memory (β = 0.40, *p* = 0.02). The association was positive in each of these cases, suggesting that performance improved with an increasing duration of pain. The contribution of chronicity was also close to significance in the hit response reaction time model (β = 0.36, *p* = 0.07), which would suggest an increase in reaction time with increased pain chronicity.

The number of painful areas was found to be an effective predictor of immediate verbal memory (story-unit recall: β = −0.34, *p* = 0.05; theme-unit recall: β = −0.37, *p* = 0.04) and delayed verbal memory (β = −0.35, *p* = 0.04), whereby “more painful sites” was associated with poorer performance. The interaction of the number of painful areas and age was a significant contributor to the model of the spatial span reverse trial (β = −0.36, *p* = 0.03, ANOVA *p* = 0.06) and the number of random responses on the CPT (β = 0.30, *p* = 0.03).

Chronic pain grade significantly predicted D-prime scores of attention (β = −0.43, *p* = 0.002), as did disability score (β = −0.28, *p* = 0.03). The interaction term of disability score by age also made a significant contribution to the memory retention model (β = −0.47, *p* = 0.006), while the interaction of present pain intensity and age had an effect close to significant on the number of hits in the CPT (β = −0.31, *p* = 0.07). The negative association indicates that worse pain was associated with a lower performance level on this task. In general, therefore, aspects of verbal memory and attention appear to be related to a variety of pain symptom and disability variables. When the total number of medications, and individual medication classifications, were entered as variables of interest, no main effects were observed on any of the cognitive outcome measures.

## Discussion

This study adds to the evidence base that chronic pain patients are impaired on memory and attention tasks; in this case, in patients with neuropathic or radicular pain. Although matched for age and education, IQ was significantly lower in neuropathic/radicular pain patients than in controls and IQ score was significantly predicted by pain in the regression model. There was a trend for pain-related deficits in reversal of spatial memory in the spatial-span task, and the pain-age interaction negatively predicted verbal memory performance. A pattern of abnormal responding on the attention task was observed in pain patients, particularly in the older group. Performance on the Wisconsin Card Sorting Task was not affected by the presence of pain or by pain**-**age interaction. Individual pain variables (number of painful areas, pain intensity, pain-related disability) were inversely correlated with cognitive performance, in particular those related to memory. Conversely, there was a positive relationship between pain duration and measures of IQ and verbal memory. The findings of the study are considered below.

### Participant characteristics

Comparison of patient and control groups revealed that the proportion of smokers was greater in pain patients than in matched healthy controls, consistent with previous findings (Ekholm et al., 2009; Zvolensky et al., 2009). However, smoking status and the time since last nicotine consumption were correlated with very few cognitive outcomes.

There were no between-group differences in the time since last caffeine consumption; however, the duration since last consumption was positively correlated with measures of attention, suggesting improved performance with increased duration since last caffeine intake. This appears to contradict the established acute effect of caffeine as a CNS stimulant. It is possible that negative effects of caffeine withdrawal on attention may have diminished with time since last consumption, or tolerance to the effects of caffeine may develop in heavy users (Fredholm et al., 1999). Regularity and average quantity of caffeine consumption was not measured in this study but should be considered for future studies.

Depressive-symptom and anxiety scores were significantly elevated in pain patients, consistent with the well-documented relationship between chronic pain and affective disorders (McCracken et al., 1996; Von Korff and Simon, 1996; Fishbain et al., 1997; Gureje et al., 1998; Raftery et al., 2011). Notably, 50% of patient participants met the PHQ diagnostic criteria for depression (compared with 0% control participants) and over 70% of patients scored higher than the standard normative scores, compared with 18% of the controls. While state anxiety score is not necessarily indicative of an anxiety disorder, these results suggest that pain patients were more susceptible to situational anxiety associated with the assessment or with their clinic visit, than were controls.

Recording medication use is important in studies examining the influence of pain on cognitive performance (McGuire, 2013). Within our patient group, five medication classifications were identified. Although anticonvulsant and antidepressant treatments were found to correlate with several cognitive measures, neither treatment significantly contributed to the overall regression models for cognitive variables. This is perhaps surprising given that analgesic agents, in particular opioids, tricyclic antidepressants, and anticonvulsants, have been linked with cognitive dysfunction and somnolence (Kerr et al., 1991; Spring et al., 1992; Raja et al., 2002; Salinsky et al., 2010). However, in the presence of chronic pain, negative effects of these treatments may be diminished, or cognitive performance may improve (Jamison et al., 2003; Kendall et al., 2010) as pain and its associated cognitive deficits are alleviated.

### Cognitive measures

In regression analysis, a significant effect of pain condition was found on general intelligence as measured by the WAIS dyad, with patients having lower IQs than controls, despite matching, and statistically accounting, for years of education. This may be further evidence of cognitive impairment in pain patients, as the WAIS subtests draw on cognitive resources such as attention and memory (Groth-Marnat, 2009).

The fact that no association was found between pain and performance on the spatial span forward task is surprising, given evidence from previous clinical (Dick and Rashiq, 2007; Luerding et al., 2008) and preclinical (Leite-Almeida et al., 2009; Hu et al., 2010; Ren et al., 2011) studies showing a negative association between neuropathic pain and spatial memory. A trend for an effect of group was observed in the reversal of spatial memory assessed in the spatial span task, with patients scoring lower than controls. The reverse spatial span task shows some conceptual similarities to the rodent Morris water maze reversal task, and deficits in this task have previously been demonstrated in a rat model of neuropathic pain (Leite-Almeida et al., 2009; Moriarty et al., 2016). A significant group-age interaction effect was observed in the assessment of verbal recognition memory, with the data indicating a pain-related effect specifically in older participants. Deficits in recognition memory have been observed previously in chronic pain (fibromyalgia) patients, independent of an interaction with age (Park et al., 2001). Recognition memory deficits have also been shown in rodent models of chronic pain (Cain et al., 1997; Lindner et al., 1999; Millecamps et al., 2004; Kodama et al., 2011). Thus, pain, or the interaction of pain with age, contributed to decreased IQ and deficits on specific subtests of memory.

There was no effect of pain condition or pain-age interaction on the D-prime measure of attention in the CPT, contrary to previous findings of impaired attention in chronic pain (Eccleston, 1994; Grisart and Plaghki, 1999; Dick et al., 2002; Veldhuijzen et al., 2006; Dick and Rashiq, 2007; Oosterman et al., 2011) and pain-related attentional deficits in rodent models of chronic pain (Cain et al., 1997; Lindner et al., 1999; Millecamps et al., 2004; Boyette-Davis et al., 2008; Pais-Vieira et al., 2009; Kodama et al., 2011). A pain-age interaction effect was observed in the model for the number of false alarms (and for number of random responses, though this was just below the level of statistical significance). There was also a significant effect of the interaction term in the model for reaction time. Younger controls had shorter reaction times than younger pain patients, but older patients were quicker to respond than older controls. Thus, older patients had increased incorrect responses and also tended to respond faster, possibly indicative of impaired inhibitory control. Impaired inhibitory control could be associated with underlying dysfunction of the prefrontal cortex (PFC), and it is known that there are morphological, neuroplastic and neurochemical changes in the PFC related to chronic pain (Moriarty et al., 2011).

The absence of any effects of pain or pain-age interaction on the WCST was unexpected given the strong body of literature to suggest that executive functions (and analogous cognitive flexibility in rodents) are impaired in chronic pain (Grisart and Van der Linden, 2001; Apkarian et al., 2004a; Mongini et al., 2005; Karp et al., 2006; Leite-Almeida et al., 2009; Verdejo-Garcia et al., 2009; Walteros et al., 2011). However, a number of previous studies failed to show an effect of pain on executive function (Suhr, 2003; Scherder et al., 2008). There is therefore a need for further research on the effect of pain and the pain-age interaction on executive functioning, preferably utilizing a wide range of executive-function measures. We hypothesize that pain related cognitive impairments may be due to structural or molecular changes that occur in the brains of chronic pain patients, and given the overlap of pain- and cognitive-associated processing regions, that this may have functional consequences that impact on cognitive performance. Specifically related to executive functioning, structural and functional changes have been observed in both chronic pain patients and in animal models of chronic pain (Apkarian et al., 2004b; Luerding et al., 2008; Metz et al., 2009; Seminowicz et al., 2009). It is possible that these changes are insufficient to produce impairments on a task such as the WCST in a robust and reliable manner. Chronic pain patients also show deficits on tasks that require rapid attentional switching (Ryan et al., 1993; Eccleston, 1994; Bosma and Kessels, 2002; Karp et al., 2006; Jongsma et al., 2011; Miro et al., 2011) and tasks involving emotional decision-making and emotional self-regulation (Apkarian et al., 2004a; Solberg Nes et al., 2009; Verdejo-Garcia et al., 2009). Performance on different tasks measuring executive-type functions may be mediated by different neuroanatomical subregions and using a single psychometric measure of performance may not be appropriate. We also noted that the overall performance of our sample (both patients and controls) on the WCST was relatively poor (scores of 89–93 compared with standard score average of 100). Thus, the present sample may have some characteristics that limit the extent to which we can generalize from these findings.

The number of painful areas reported was a predictor of verbal memory, with scores decreasing as pain diffuseness increased. Pain chronicity was found to be a positive predictor of IQ and verbal memory. Some studies have shown that pain chronicity was not associated with differences in cognitive function (Eccleston, 1994; Alanoglu et al., 2005), whereas others have shown that duration of a painful illness was inversely related to cognitive performance (Calandre et al., 2002; Apkarian et al., 2004a; Ryan, 2005; Verdejo-Garcia et al., 2009). A positive correlation, as observed herein, has not been demonstrated previously, but may represent a habituation or compensation mechanism induced in prolonged pain states. Further investigation of this theory is warranted. Previous reports of an inverse correlation included chronic migraine and diabetic patients (Calandre et al., 2002; Ryan, 2005). In the context of such illnesses, it is important to note that there may be a cumulative effect of disease duration and that the relationship between pain chronicity and cognitive function may be confounded by additional factors. A study by Seminowicz et al. (2011) showed, using functional imaging, that the dorsolateral PFC in chronic pain patients was thinner and abnormally activated during a cognitive task. However, following effective analgesic treatment, this region showed increased thickness and task-related activity patterns were normalized. These results suggest that pain-related cognitive impairment may be reversible with treatment over time. Thus, there may be a highly complex relationship between duration of pain, analgesic treatment and cognitive-task performance, an investigation of which was beyond the scope of the present study. There is also emerging evidence of positive adaptive plasticity that may derive from psychological interventions for chronic pain or use of specific coping strategies (see for example, Braden et al., 2016; Lazaridou et al., 2017; Malfliet et al., 2017) so that improvements in these psychological processes over time may explain a decreasing effect of pain despite increasing chronicity.

Pain intensity was not generally associated with alterations in cognitive function, which suggests that other aspects of the pain experience may contribute to changes in cognition. Chronic pain grade and pain-related disability negatively affected aspects of attention, suggesting that this cognitive domain may be more susceptible to the affective or disabling dimensions of pain. Interactions between pain variables and age also made contributions to memory and attention outcomes, again indicating an important interplay between these variables.

### Limitations

A sample size of 76 could be considered relatively small, however, sample sizes of this order have been used previously in pain-cognition studies (Eccleston, 1994; Grace et al., 1999; Grisart and Plaghki, 1999; Park et al., 2001; Dick et al., 2002; Apkarian et al., 2004a; Harman and Ruyak, 2005; Mongini et al., 2005; Karp et al., 2006; Dick and Rashiq, 2007; Rodriguez-Andreu et al., 2009; Verdejo-Garcia et al., 2009; Walteros et al., 2011). The pain variable measures and the records of current medication use relate only to the patient subset of participants and so sample size is further reduced for these measures (*n* = 38). Therefore, effects of medication on cognitive outcomes cannot be ruled out completely, notwithstanding the application of appropriate statistical controls. The apparent clinical under-recognition of depression within the present sample of pain patients means that the effects of depression could not be controlled for experimentally. Depressive symptoms was strongly correlated with presence of pain and was also significantly correlated with a number of cognitive outcomes. In spite of this, significant contributions of pain and pain-age interaction to some cognitive scores were found, over and above the contribution of depression. It is unlikely that the study is compromised by the presence of undiagnosed depression, since an increased prevalence of depression is known to be associated with chronic pain (Von Korff and Simon, 1996; Raftery et al., 2011). Finally, it is not known whether this series of tasks has been used in combination previously, it is possible that the order in which the tests were administered may have affected task performance. Motivation may affect performance of attentional tasks in chronic pain (Keogh et al., 2013) and was not measured here, but no obvious decrease in participant effort or motivation was observed during the assessment.

## Conclusions

This study provides further support for the theory that pain affects cognition and that the relationship is influenced by age. The cognitive outcomes affected were mainly within the domains of memory and attention, with IQ and psychomotor speed also affected. Further research is required to determine whether the present set of outcomes represents a specific signature of cognitive performance in neuropathic and radicular pain.

## Ethics statement

This study was carried out in accordance with the recommendations of The Research Ethics Committees of Galway University Hospital and the National University of Ireland Galway with written informed consent from all subjects. All subjects gave written informed consent in accordance with the Declaration of Helsinki. The protocol was approved by The Research Ethics Committees of Galway University Hospital and the National University of Ireland Galway.

## Author contributions

All authors listed, have made substantial, direct and intellectual contribution to the work, and approved it for publication.

### Conflict of interest statement

The authors declare that the research was conducted in the absence of any commercial or financial relationships that could be construed as a potential conflict of interest.
