# Supplementary material for: Cognitive Impairment in Patients with Chronic Neuropathic or Radicular Pain: An Interaction of Pain and Age
Source: Front Behav Neurosci. 2017 Jun 13;11:100. doi: 10.3389/fnbeh.2017.00100 (PMC5468384; doi:10.3389/fnbeh.2017.00100)
Supplement: Supplementary file 1 [file DataSheet1.docx]

Table S1: Bivariate correlation matrix of participant characteristics and cognitive outcomes – r correlation coefficients

|  | **Group** | **Age** | **Gender** | **Years of Education** | **Smoker** | **Time since last nicotine** | **Time since last caffeine** | **Time since last alcohol** | **Depression score** | **State anxiety score** |
| --- | --- | --- | --- | --- | --- | --- | --- | --- | --- | --- |
| **Participant characteristics** |  |  |  |  |  |  |  |  |  |  |
| Group | 1 |  |  |  |  |  |  |  |  |  |
| Age | −0.08 | 1 |  |  |  |  |  |  |  |  |
| Gender | 0.00 | −0.01 | 1 |  |  |  |  |  |  |  |
| Years of Education | 0.21 | −0.27^*^ | 0.04 | 1 |  |  |  |  |  |  |
| Smoker classification | 0.33^**^ | 0.07 | 0.06 | 0.20 | 1 |  |  |  |  |  |
| Time since last nicotine | 0.03 | −0.35 | −0.05 | −0.10 | 0.28 | 1 |  |  |  |  |
| Time since last caffeine | 0.10 | −0.40^**^ | 0.17 | −0.07 | 0.10 | 0.42^*^ | 1 |  |  |  |
| Time since last alcohol | −0.28^*^ | 0.084 | 0.03 | −0.13 | −0.33^**^ | 0.08 | −0.02 | 1 |  |  |
| Depression score | −0.84^**^ | −0.01 | −0.07 | −0.26^*^ | −0.27^*^ | 0.19 | −0.07 | 0.23 | 1 |  |
| State anxiety score | −0.59^**^ | −0.03 | −0.06 | −0.36^**^ | −0.09 | 0.37 | 0.08 | 0.21 | 0.69^**^ | 1 |
| **Cognitive variables** |  |  |  |  |  |  |  |  |  |  |
| Estimated FSIQ | 0.49^**^ | −0.27^*^ | 0.01 | 0.52^**^ | 0.21 | 0.10 | 0.06 | −0.17 | −0.47^**^ | −0.42^**^ |
| **Immediate Verbal Memory**: |  |  |  |  |  |  |  |  |  |  |
| Story Unit Recall | 0.46^**^ | −0.13 | 0.26^*^ | 0.28^*^ | 0.28^*^ | 0.25 | 0.10 | −0.25^*^ | −0.45^**^ | −0.32^**^ |
| Theme Unit Recall | 0.39^**^ | −0.07 | 0.17 | 0.19 | 0.26^*^ | 0.00 | −0.04 | −0.22 | −0.36^**^ | −0.21 |
| Learning slope | 0.17 | −0.28^*^ | −0.07 | 0.24^*^ | −0.01 | 0.04 | 0.09 | −0.11 | −0.11 | −0.24^*^ |
| **Delayed Verbal Memory:** |  |  |  |  |  |  |  |  |  |  |
| Unit Recall | 0.36^**^ | −0.13 | 0.10 | 0.32^**^ | 0.13 | 0.20 | −0.05 | −0.22 | −0.37^**^ | −0.29^*^ |
| Theme Recall | 0.22 | −0.15 | 0.14 | 0.16 | 0.13 | −0.07 | 0.03 | −0.30^*^ | −0.19 | −0.13 |
| Recognition | 0.17 | −0.19 | 0.15 | 0.21 | 0.21 | 0.19 | −0.01 | −0.13 | −0.22 | −0.23^*^ |
| % Retention | −0.02 | −0.20 | −0.12 | 0.21 | −0.16 | 0.05 | −0.20 | −0.03 | 0.02 | −0.10 |
| **Spatial Memory:** |  |  |  |  |  |  |  |  |  |  |
| Forward | 0.23^*^ | −0.12 | 0.03 | 0.11 | 0.17 | −0.15 | 0.17 | −0.15 | −0.25^*^ | −0.24^*^ |
| Reverse | 0.24^*^ | 0.07 | −0.14 | 0.23^*^ | 0.01 | −0.04 | −0.05 | −0.23 | −0.22 | −0.22 |
| Total | 0.27^*^ | −0.02 | −0.07 | 0.18 | 0.12 | −0.05 | 0.05 | −0.21 | −0.24^*^ | −0.22 |

Table S1: continued from previous page

|  | **Group** | **Age** | **Gender** | **Years of Education** | **Smoker** | **Time since last nicotine** | **Time since last caffeine** | **Time since last alcohol** | **Depression score** | **State anxiety score** |
| --- | --- | --- | --- | --- | --- | --- | --- | --- | --- | --- |
| **Attention:** |  |  |  |  |  |  |  |  |  |  |
| Hits | 0.17 | −0.34^**^ | −0.05 | 0.24^*^ | −0.02 | 0.08 | 0.11 | 0.01 | −0.31^**^ | −0.21 |
| False alarms | −0.17 | 0.35^**^ | −0.04 | −0.34^**^ | 0.07 | −0.33 | −0.30^*^ | 0.12 | 0.16 | 0.06 |
| Randoms | −0.16 | 0.35^**^ | 0.03 | −0.36^**^ | 0.01 | −0.17 | −0.16 | 0.06 | 0.14 | 0.18 |
| D-Prime | 0.23^*^ | −0.45^**^ | 0.00 | 0.41^**^ | −0.06 | 0.31 | 0.24^*^ | −0.10 | −0.28^*^ | −0.16 |
| T-score | 0.22 | −0.44^**^ | −0.01 | 0.39^**^ | −0.08 | 0.36 | 0.25^*^ | −0.09 | −0.29^*^ | −0.23^*^ |
| T-score corrected | 0.18 | −0.29^*^ | 0.13 | 0.35^**^ | −0.08 | 0.27 | 0.19 | 0.00 | −0.30^*^ | −0.27^*^ |
| **Psychomotor Speed:** |  |  |  |  |  |  |  |  |  |  |
| Hit reaction time | 0.11 | 0.15 | 0.25^*^ | −0.09 | 0.13 | 0.12 | −0.02 | −0.07 | −0.07 | 0.04 |
| False Alarm reaction time | 0.01 | 0.02 | 0.23^*^ | 0.08 | 0.11 | 0.25 | 0.05 | −0.07 | 0.09 | 0.14 |
| **Executive Function:** |  |  |  |  |  |  |  |  |  |  |
| Errors | 0.15 | −0.17 | 0.11 | −0.04 | −0.02 | 0.11 | 0.24^*^ | −0.16 | −0.13 | −0.11 |
| Perseverative responses | 0.13 | −0.07 | 0.17 | −0.20 | −0.03 | 0.13 | 0.24^*^ | −0.13 | −0.15 | −0.01 |
| Perseverative errors | 0.16 | −0.08 | 0.17 | −0.18 | −0.03 | 0.12 | 0.23 | −0.15 | −0.16 | −0.03 |
| Non-perseverative errors | 0.07 | −0.24^*^ | 0.07 | 0.03 | −0.01 | 0.06 | 0.20 | −0.10 | −0.04 | −0.17 |
| Conceptual level responses | 0.18 | −0.19 | 0.09 | −0.01 | 0.02 | 0.06 | 0.24^*^ | −0.16 | −0.14 | −0.12 |
| Categories completed | 0.20 | −0.29^*^ | −0.01 | 0.26^*^ | 0.00 | 0.07 | 0.19 | −0.18 | −0.24^*^ | −0.21 |
| Trials to 1^st^ category | −0.14 | 0.17 | −0.09 | −0.15 | 0.06 | 0.15 | −0.27^*^ | 0.00 | 0.19 | 0.27^*^ |
| Failure to maintain set | −0.20 | 0.19 | 0.11 | −0.06 | −0.10 | 0.26 | −0.15 | 0.35^**^ | 0.27^*^ | 0.13 |
| Learning to learn | 0.06 | −0.19 | 0.09 | −0.03 | −0.09 | 0.21 | −0.16 | −0.11 | 0.05 | 0.13 |
| **p* < 0.05, ***p* < 0.01 | | | | | | | | | | |
