# Supplementary material for: Cognitive Impairment in Patients with Chronic Neuropathic or Radicular Pain: An Interaction of Pain and Age
Source: Front Behav Neurosci. 2017 Jun 13;11:100. doi: 10.3389/fnbeh.2017.00100 (PMC5468384; doi:10.3389/fnbeh.2017.00100)
Supplement: Supplementary file 3 [file DataSheet3.docx]

Table S3: Correlation matrix of patient-specific characteristics and cognitive outcomes – r correlation coefficients

|  | **Medication** | **Opioid** | **Anti-convulsant** | **Anti-depressant** | **NSAID** | **Other** | **Number of medications** | **% Pain relief** | **Self-assessed cognition** |
| --- | --- | --- | --- | --- | --- | --- | --- | --- | --- |
| Estimated FSIQ | −0.08 | 0.12 | −0.01 | −0.24 | −0.02 | −0.12 | 0.06 | −0.04 | −0.16 |
| **Immediate Verbal Memory**: |  |  |  |  |  |  |  |  |  |
| Unit Recall | −0.15 | 0.12 | 0.15 | −0.09 | −0.07 | −0.12 | 0.08 | −0.13 | −0.26 |
| Theme Recall | −0.02 | 0.10 | 0.06 | 0.03 | −0.19 | 0.13 | −0.11 | −0.21 | −0.07 |
| Learning slope | −0.22 | 0.15 | 0.37^*^ | 0.01 | 0.03 | −0.11 | −0.14 | −0.06 | −0.06 |
|  |  |  |  |  |  |  |  |  |  |
| **Delayed Verbal Memory:** |  |  |  |  |  |  |  |  |  |
| Unit Recall | −0.18 | 0.12 | 0.25 | −0.03 | 0.03 | −0.05 | −0.04 | −0.17 | −0.07 |
| Theme Recall | −0.19 | 0.12 | 0.17 | −0.02 | −0.13 | 0.13 | −0.11 | −0.22 | −0.08 |
| Recognition | 0.06 | −0.08 | −0.08 | −0.29 | −0.28 | −0.21 | 0.19 | 0.05 | −0.10 |
| % Retention | 0.07 | −0.14 | 0.11 | 0.06 | 0.14 | 0.09 | −0.06 | 0.05 | 0.25 |
| **Spatial Memory:** |  |  |  |  |  |  |  |  |  |
| Forward | −0.19 | 0.13 | 0.01 | 0.22 | 0.08 | −0.03 | −0.27 | −0.14 | −0.10 |
| Reverse | −0.25 | 0.22 | 0.00 | −0.10 | 0.02 | −0.13 | 0.02 | −0.21 | −0.18 |
| Total | −0.25 | 0.21 | 0.01 | 0.02 | 0.01 | −0.08 | −0.13 | −0.25 | −0.06 |
| **Attention:** |  |  |  |  |  |  |  |  |  |
| Hits | −0.06 | 0.00 | 0.18 | −0.20 | 0.09 | 0.18 | −0.04 | 0.01 | −0.29 |
| False alarms | −0.27 | 0.26 | −0.05 | 0.30 | −0.02 | 0.30 | −0.26 | 0.10 | 0.05 |
| Randoms | −0.24 | 0.11 | 0.10 | 0.29 | 0.28 | 0.13 | −0.25 | 0.00 | 0.12 |
| D-Prime | 0.17 | −0.17 | 0.11 | −0.38^*^ | 0.06 | −0.17 | 0.14 | −0.14 | −0.18 |
| T-score | 0.06 | −0.13 | 0.11 | −0.30 | 0.11 | −0.05 | 0.08 | −0.17 | −0.18 |
| T-score corrected | 0.08 | −0.15 | 0.04 | −0.35^*^ | 0.11 | −0.01 | 0.11 | −0.11 | −0.14 |
| **Psychomotor Speed:** |  |  |  |  |  |  |  |  |  |
| Hit reaction time | 0.21 | −0.02 | 0.05 | −0.15 | −0.02 | −0.13 | 0.22 | −0.06 | −0.02 |
| False Alarm reaction time | 0.26 | 0.00 | 0.05 | −0.09 | −0.04 | −0.15 | 0.22 | −0.13 | 0.21 |

Table S3: continued from previous page.

|  | **Medication** | **Opioid** | **Anti-convulsant** | **Anti-depressant** | **NSAID** | **Other** | **Number of medications** | **% Pain relief** | **Self-assessed cognition** |
| --- | --- | --- | --- | --- | --- | --- | --- | --- | --- |
| **Executive Function:** |  |  |  |  |  |  |  |  |  |
| Errors | −0.19 | 0.27 | 0.12 | −0.27 | 0.08 | −0.03 | −0.01 | −0.25 | −0.03 |
| Perseverative responses | −0.32^*^ | 0.31 | 0.10 | −0.14 | 0.01 | 0.12 | −0.13 | −0.22 | −0.01 |
| Perseverative errors | −0.31 | 0.30 | 0.10 | −0.14 | 0.01 | 0.14 | −0.14 | −0.24 | 0.01 |
| Non-perseverative errors | 0.01 | 0.04 | 0.22 | −0.22 | 0.20 | −0.25 | 0.10 | −0.11 | −0.06 |
| Conceptual level responses | −0.18 | 0.27 | 0.12 | −0.29 | 0.06 | −0.06 | 0.02 | −0.25 | −0.04 |
| Categories completed | −0.07 | −0.01 | 0.00 | −0.29 | −0.06 | −0.05 | 0.09 | −0.12 | −0.24 |
| Trials to 1^st^ category | 0.07 | −0.06 | −0.03 | 0.34^*^ | −0.15 | 0.14 | −0.15 | 0.07 | −0.04 |
| Failure to maintain set | 0.09 | 0.01 | −0.09 | −0.23 | −0.10 | −0.05 | 0.18 | −0.09 | 0.45^**^ |
| Learning to learn | 0.04 | −0.22 | 0.00 | 0.14 | −0.24 | 0.28 | −0.10 | −0.01 | −0.04 |
| **p* < 0.05, ***p* < 0.01 | | | | | | | | | |
